# Dynamics of crime activities in the network of city community areas

**DOI:** 10.1007/s41109-019-0239-8

**Published:** 2019-12-26

**Authors:** Xiang Niu, Amr Elsisy, Noemi Derzsy, Boleslaw K. Szymanski

**Affiliations:** 1 Network Science and Technology Center & Department of Computer Science, Rensselaer Polytechnic Institute, 110 8th St, Troy, NY USA; 2 Network Science and Technology Center & Department of Physics, Rensselaer Polytechnic Institute, 110 8th St, Troy, NY USA

## Abstract

Understanding criminal activities, their structure and dynamics are fundamental for designing tools for crime prediction that can also guide crime prevention. Here, we study crimes committed in city community areas based on police crime reports and demographic data for the City of Chicago collected over 16 consecutive years. Our goal is to understand how the network of city community areas shapes dynamics of criminal offenses and demographic characteristics of their inhabitants. Our results reveal the presence of criminal hot-spots and expose the dynamic nature of criminal activities. We identify the most influential features for forecasting the per capita crime rate in each community. Our results indicate that city community crime is driven by spatio-temporal dynamics since the number of crimes committed in the past among the spatial neighbors of each community area and in the community itself are the most important features in our predictive models. Moreover, certain urban characteristics appear to act as triggers for the spatial spreading of criminal activities. Using the k-Means clustering algorithm, we obtained three clearly separated clusters of community areas, each with different levels of crimes and unique demographic characteristics of the district’s inhabitants. Further, we demonstrate that crime predictive models incorporating both demographic characteristics of a community and its crime rate perform better than models relying only on one type of features. We develop predictive algorithms to forecast the number of future crimes in city community areas over the periods of one-month and one-year using varying sets of features. For one-month predictions using just the number of prior incidents as a feature, the critical length of historical data, *τ*_*c*_, of 12 months arises. Using more than *τ*_*c*_ months ensures high accuracy of prediction, while using fewer months negatively impacts prediction quality. Using features based on demographic characteristics of the district’s inhabitants weakens this impact somewhat. We also forecast the number of crimes in each community area in the given year. Then, we study in which community area and over what period an increase in crime reduction funding in this area will yield the largest reduction of the crime in the entire city. Finally, we study and compare the performance of various supervised machine learning algorithms classifying reported crime incidents into the correct crime category. Using the temporal patterns of various crime categories improves the classification accuracy. The methodologies introduced here are general and can be applied to other cities for which data about criminal activities and demographics are available.

## Introduction

Criminal activities have been extensively studied for decades by sociologists, criminologists and law enforcement agents in a continuous effort to reduce crime in cities (Bettencourt 2015; Griffiths and Chavez 2004; Guarino-Ghezzi and Trevino 2010). In the 21^*st*^ century this problem has been gaining ever increasing importance and attention in the context of a vision for smart cities urban development. However, crime reduction and prevention require deep understanding of the structure and dynamics of crime, which due to its complex nature is difficult to achieve (D’Orsogna and Perc 2015). The advancement of computational tools and the increasing availability of real data allow researchers to improve the study, understanding and modeling of criminal activities. Recently, several cities across the U.S. have made their crime records publicly available. Such data release benefits scientists by providing them with the access to data that can lead to effective crime modeling and forecasting, to help law enforcement agencies to better understand criminal activities, and to optimally allocate additional funding as preventive measures aiming to reduce crime.

Recent studies performed on crime data released by the government agencies have shown that these crimes do not occur in isolation. Instead they exhibit spatio-temporal dynamics in city community areas (Alves et al. 2015; Anselin et al. 2000; Backstrom et al. 2010; Gordon 2010; Murray and Grubesic 2013; Oliveira et al. 2018; Zeoli et al. 2014). In addition, the levels of crimes at community level have been shown to be strongly correlated with demographic features (Alves et al. 2013; Alves et al. 2018; da Cunha and Gonçalves 2018). Moreover, certain urban characteristics such as importance of community size, has been recognized as triggers for the spatial spreading of criminal activities (Furtado et al. 2007). Also, it has been demonstrated here and in (Almanie et al. 2015) that incorporating into the crime predictive models both demographic and spatial information increases their predictive capabilities.

Here, we study dynamics of criminal activities in city community areas based on police crime reports and demographic data collected for the City of Chicago for 16 consecutive years. We also introduce the predictive algorithms aiming at forecasting monthly and yearly criminal activities in the Chicago community areas. Finally, we discuss how to choose a community area and time period over which to deploy additional crime reduction funding to optimize the crime reduction in the entire city.

## Patterns of criminal activities in city community areas

We start by analyzing the patterns of criminal activities in city community areas and investigate their relationship to their inhabitants’ demographic data extracted from the census data.

### Datasets

We collected and analyzed here the public crime records for the 16 consecutive years (2002 to 2017) and the census data for the 18 consecutive years (2000 to 2017) in the City of Chicago. For administrative purposes, the City of Chicago is split into 50 wards that correspond in total to 77 community areas, to which we will refer in short as communities. It’s important to note that crime data was reported yearly, while census data was reported only for the years 2000, 2008, 2012, 2013, and 2017. Therefore we interpolate the census data from the reported years to obtain reported or interpolated data for each year from 2001 to 2017.

Here, we focus on the study of crime dynamics at the level of community, for each of which we extract demographic information from the census data provided by the U.S. Census Bureau (U.S. Census Bureau). We extract also crime information from the crime incident reports obtained from The City of Chicago Data Portal, records extracted from the Chicago Police Department’s CLEAR (Citizen Law Enforcement Analysis and Reporting) system (Public Safety Data), and the IUCR (Illinois Uniform Crime Reporting) codes (Chicago Police Department). We limit our collection of data and its analysis to records of violent crimes as identified by IUCR (i.e., burglary, assault, homicide).

### Criminal hot-spots

**Fig. 1:**
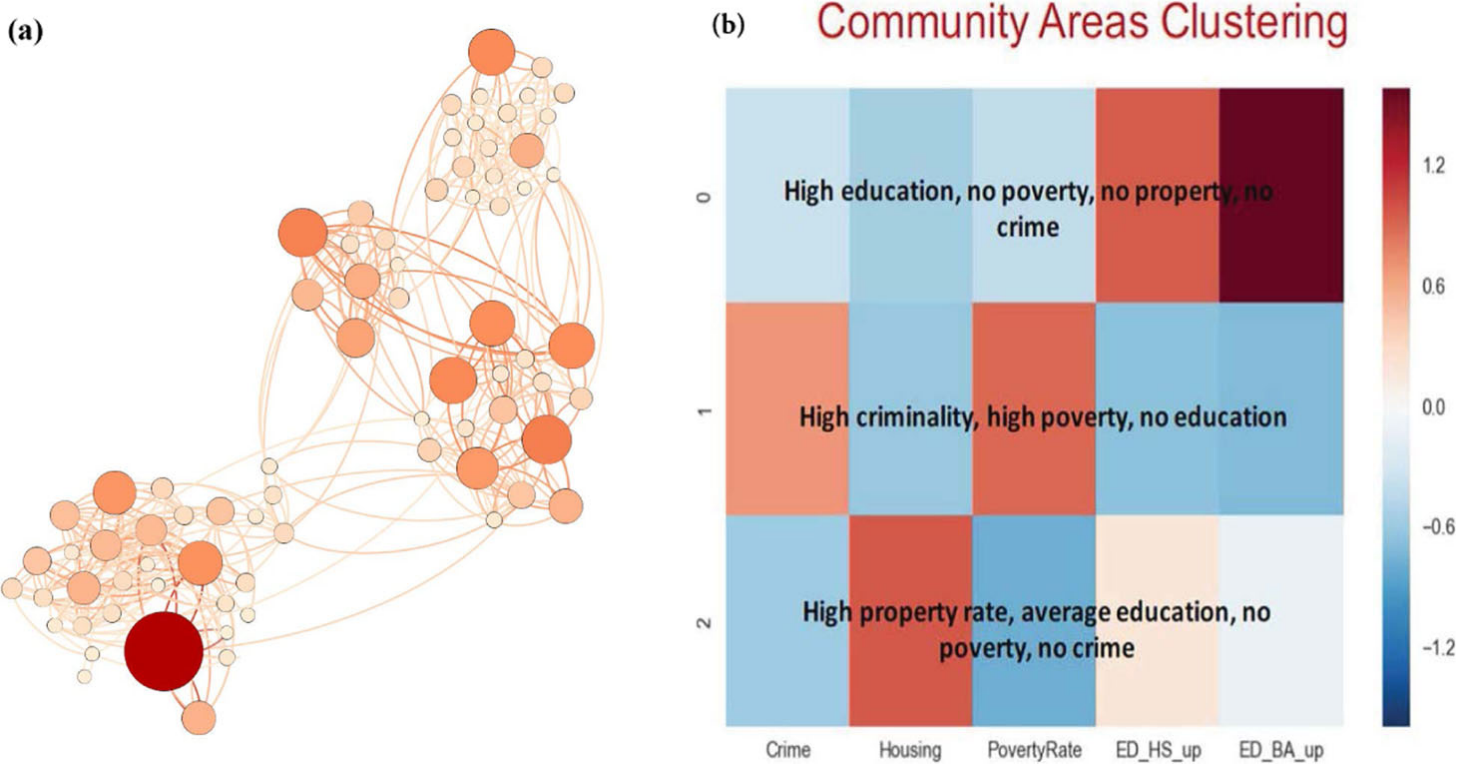
Network of Chicago 77 communities. (**a**) Community graph for year 2017. There are 77 nodes representing communities and 204 edges connecting a pair of communities sharing a boundary with each other. The average degree is 5.3 and diameter 9. The entire network consists of one fully connected component. The darkness of color is proportional to the crime rate and size is proportional to population of each cluster. The clusters of high crime are clearly visible. (**b**) Three clusters of communities. Each cluster contains communities with the same range of values for three features: level of poverty, percentage of property ownership, and level of education

High frequency of criminal activities often occurs in spatially localized communities called criminal hot-spots (Oliveira et al. 2017; Short et al. 2010; Weisburd 2015). To verify that this is true in Chicago, we first define a hot-spot as a community area in which the crimes rate for the given year exceed the average crime rate by at least 1 and 1/2 of the standard deviation. This means that only 6.7% of all community areas will qualify each year, so from 4 to 6 communities. Then, we create a graph, shown in Fig. 1a in which nodes represent communities and undirected edges connect each pair of communities that share a common boundary. Analyzing this graph, we find that some but not all these hot-spots occur in community areas that are clustered together into a traditional community (cluster) of community areas. This cluster has density of edges inside it higher than across it, as require by the modularity metric for such clusters (Newman 2006). As observed in Table 1, such is a cluster of community areas 37, 40 and 68 forming full triangle and therefore present (in different positions, from 2002 to 2015 in the hot-spot list. As seen in this table, the first position of occupied over all 16 year by just two communities, 32 and 37, while the 5th position is occupied by six communities, 26, 38, 40, 44, 67 and 68.

**Table 1:** For all years of data, we look at communities with crime rate that exceeds the average crime rate by at least 1 and 1/2 of the standard deviation for that year

year	1st		2nd		3rd		4th		5th		6th	
	Id	rate	id	rate	Id	rate	Id	rate	Id	rate	Id	rate
2002	37	0.158	32	0.142	40	0.111	68	0.107	38	0.097		
2003	37	0.184	32	0.177	40	0.147	44	0.132	68	0.128	67	0.126
2004	32	0.185	37	0.180	40	0.143	68	0.129	44	0.125	69	0.121
2005	32	0.175	37	0.174	40	0.143	68	0.127	44	0.122	67	0.120
2006	37	0.175	32	0.175	40	0.152	68	0.132	67	0.122	26	0.121
2007	32	0.177	68	0.142	40	0.140	37	0.140	67	0.128		
2008	32	0.187	37	0.173	68	0.145	67	0.129	40	0.120		
2009	32	0.168	37	0.135	68	0.133	40	0.120	67	0.117	69	0.106
2010	32	0.161	37	0.158	68	0.129	40	0.122	67	0.117		
2011	37	0.190	32	0.153	40	0.124	68	0.122	67	0.116		
2012	37	0.171	32	0.154	40	0.114	68	0.113	26	0.106	67	0.102
2013	32	0.142	37	0.137	40	0.110	68	0.100	67	0.095	44	0.090
2014	32	0.134	37	0.127	40	0.095	68	0.091	44	0.082		
2015	32	0.144	37	0.130	40	0.098	68	0.084				
2016	37	0.172	32	0.163	26	0.096	40	0.094				
2017	32	0.187	37	0.170	26	0.098	40	0.094				

This results suggest the importance of spatial proximity information, such as the crime lever and/or demographics characteristics of community areas that are neighbors of (or in other words are connected in the graph to) the currently analyzed community. We also observe that throughout the analyzed 16 consecutive years these hot-spots are visible every year, but some of them shift around the city. Moreover, some areas that had previously experienced elevated number of crime incidents became safer, and certain community areas with initially low number of crime incidents became less safe. Moreover, the features representing the past crime rates for the community (a temporal dimension) and the past numbers of crimes in the communities that are its geographical neighbors (a spatial dimension) are the strongest feature for prediction of future crime. This implies a dynamic nature of criminal activities, emphasizing the importance of studying criminal activities as a social contagion epidemic spreading in time and space (Zeoli et al. 2014). The specific impact of crime change in a community on neighboring communities is define by Eq. (6). As shown in the subsequent sections, the strongest two features for predicting future crime rates represent the past crime rates for the community (a temporal dimension) and the past rates of crimes in the communities that are its geographical neighbors (a spatial dimension).

Using the k-Means clustering algorithm, we obtained three clearly separated clusters of communities shown in Fig. 1b. Each cluster contains communities with the same range of values for three features: level of poverty, percentage of property ownership, and level of education. The first is a cluster characterized by low criminal activity, no poverty, and high property ownership. The second cluster contains community areas with low criminal activities, very high level of education, no poverty, and no property ownership. The last cluster comprises community areas with high criminal activities, high poverty rate, low level of property ownership, and low education. We also find that these clusters persist throughout all 16 years for which we have available data.

**Fig. 2:**
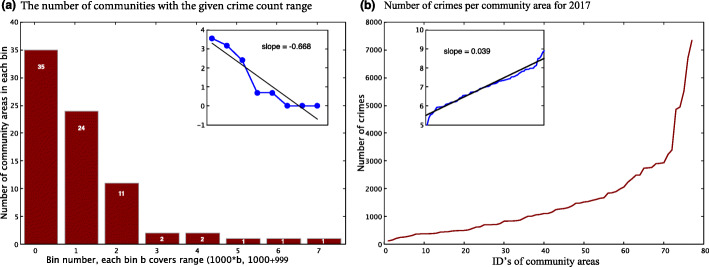
Distribution of the number of crime incidents among communities. **a** Histogram of this distribution showing the number of areas within eight disjoint bins for the number of crime incidents, where range for bin *b*=0,1…,6,7 is [*b*∗1000,*b*∗1000+999]. The inset shows that the number of communities with ever growing number of incidents decays exponentially with an exponent of -0.668 in a Zipf’s Law type manner. **b** The number of crime incidents in community areas listed in the increasing order of the number of incidents that they experienced in year 2017. The inset shows that the number of incidents follows the Power Law with the exponent of 0.039, confirming findings in (Alves et al. 2013; Green et al. 2017)

For each community, we calculate the number crimes committed within the studied time period. Next, we calculate the overall distribution of crime incidents that occurred in the city over the same period. Based on this, we consider community areas that have number of crimes above the interquartile range as *high crime community areas*, whereas communities with criminal activity levels below the interquartile range as *low crime community areas*. For the data for year 2017, shown in Fig. 2, the numbers of crime incidents in that year in the bottom 19 lowest crime communities vary from low 124 to high 498. In contrast, in the top 19 highest crime communities the lowest number of crime incidents is 1992, while the highest is 7342, so nearly fifteen times higher than in the 19 lowest crime community areas. As expected based on (Furtado et al. 2007), the distribution of the number of crime incidents in community areas decays in the Zipf’s Law type manner. One consequence of this is that more than half of the crime incidents that happened in 2017 in Chicago were located in just the top 16 highest crime community areas, while the remaining less than half incidents happened in 61 communities.

## Crime prediction

The discovery of criminal activity patterns and their influencing factors in community areas is the first stage into gaining deeper understanding of criminal behavior in city communities. The ultimate goal is to build predictive algorithms that are capable of forecasting future criminal activities with high accuracy.

### Predicting the number of crime incidents in community areas

We utilize and extend a Spatio-Temporal linear regression algorithm (Misyrlis et al. 2017) to forecast the number of crime incidents in city communities for 1 month time window of future, using prior months of historical data on criminal activities. We refer to the extent of time for which we perform the prediction in the future as future time window, and to the extent of time of historical data used in regression. We used all the spatio-temporal information available from the data, namely Date, CommunityID, ranging from 1 to 77, and NumberofIncidents, value that we computed for each period and location of interest. The implementation of the algorithm is as follows. Let *a*_*i,t*_ be the number of crime incidents at location *i* at time *t*, and *v*_*i,j*,*t*_ be the value of a feature *j* at time *t* and location *i*, with *v*_*i*,1,*t*_=*a*_*i,t*_. In order to predict the number of crime incidents at some future time *t* at each location *i* we use a vector $\vec {y}_{t}=\{a_{1,t}, a_{2,t},..., a_{i,t},\ldots, a_{77,t}\}\in R^{L}$, and the input matrix *X*_*t*_∈*R*^*L*×*τ*×*n*^, where *L*=77 denotes the total number of communities, *n* is the number of features which for basic algorithm is *n*=1 (this feature represents the number of crime incidents), while *τ* is the length of historical data. Each feature value is defined at certain location *i* and historical time instance *t*. Hence, each value of the input matrix at current time of prediction *t*_*p*_ is defined as $X_{t_{p}} = (v_{i,j,t}), i\in [1,L], j\in [1,n], t\in [t_{p}-\tau +1, t_{p}]$. In the spatio-temporal linear regression model (Misyrlis et al. 2017), at current time *t*_*p*_ (so we know data up to this time) we have

1 $$ \vec{y}_{t_{p}+1} = w X_{t_{p}}.  $$

To obtain the optimal matrix *w*, we use regression on known past data about crime and features values using the following equation

2 $$ w = \operatorname*{argmin}_{w} ||\vec{y}_{t_{p}}-wX_{t_{p}-1}||^{2}_{1+\tau\times n}.   $$

**Fig. 3:**
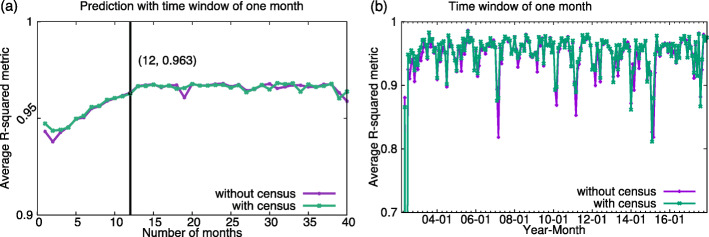
Quality of prediction of the numbers of crimes. **a** Impact of the size of historical data. The plot shows the crime prediction accuracy for future time window size of one month as a function of the various lengths of historical data. The prediction accuracy improves with the increasing size of data the most for short historical data without census information. The improvement is smaller for longer historical data. With the length of historical data of 12 months the R-squared reached 0.960 from the bottom of 0.379, and it reached the highest level at 24 month length of historical data with R-squared of 0.9680. With census data, the bottom of 0.9436 was reached with the length of historical data at 2 months, and the peak was 0.9682 with that length at 31 months. **b** Impact of the census data. The plot shows the accuracy of predictions with the length of historical data of 1 month and with and without census data. The fluctuations of performance are smaller for the predictions with census information regardless of the length of historical data

In Fig. 3a a critical time *τ*_*c*_ = 12 months can be observed since accuracy of number of crime incidents prediction of our basic algorithm constantly grows when the length of historical data is growing towards *τ*_*c*_ and it saturates for the lengths higher than this critical time. Figure 3b shows that incorporating into the historical data demographic characteristics of the community’s inhabitants of each community area abates the impact of the short length of historical data but does not eliminate the critical points existence. Such enhanced algorithm increases the prediction accuracy when the historical data is shorter than the critical time to levels similar to that seen beyond *τ*_*c*_. However, for historical data longer than the critical time, the census data improves the prediction accuracy only marginally.

The incorporated demographics of the community’s inhabitants include the following 10 features: yearly information on HomeOwnershipRate, PovertyRate, HighschoolDegreeRate, BachelorDegreeRate, and information on the racial makeup of the population living in the community areas: Total, Asian, Black, Hispanic/Latino, White, and Other. Adding these features increases the number of features from *n*=1 to *n*=11 in the definition of multi-dimensional matrix *v*_*i,j*,*t*_∈*R*^*L*×*τ*×*n*^.

To evaluate usefulness of features in explaining crime level variance, we inspect *P*-values and the standardized feature coefficients in linear regression model. The smaller *P*-value is the stronger is the evidence against the null hypothesis (of no effect). Typically, a feature with *P*-value of 0.05 or below is considered useful. The most important features identified that way for model using 1 month old historical data is the crime data for the only month of historical data available, with *P*-value of 0, and the standardized coefficient of 0.901. Then, three demographic characteristics of the community’s inhabitants for that month follow, BachelorDegreeRate (*P*-value of 0.02, the standardized coefficient of 0.070), HighschoolDegreeRate, (*P*-value of 0.013, the standardized coefficient of −0.047), and PovertyRate (*P*-value of 0.04, the standardized coefficient of 0.021). For model using previous 12 months of historical data, census data ceased to be of importance. Instead, among the previous 12 months of crime data available, the significant features listed in the order of decreasing influence are crime data for 1^*st*^,2^*nd*^, 10^*th*^, 11^*th*^, 8^*th*^, 5^*th*^, 3^*rd*^, 6^*th*^ and 9^*th*^ preceding months with small *P*-values ranging from 0.000009 to 0.04 and the absolute values of standardized coefficients ranging from 0.58 to 0.05.

To eliminate autocorrelation, we start with the true value $y_{t_{p}}$ and represent it as the sum of the one-month prediction $y^{\prime }_{t_{p}}$ and the error $e_{t_{p}}$,

3 $$ y_{t_{p}} = y'_{t_{p}}+e_{t_{p}} = w X_{t_{p}-1} + e_{t_{p}}   $$

Then,

4 $$ e_{t_{p}} = y_{t_{p}} - w X_{t_{p}-1} = \rho_{0} + \sum^{d}_{i=1} \rho_{i}e_{t_{p}-i}   $$

where *d*=12 is the time depth of the historical data used.

In the time-series regressions using only 1 month length of historical data, the error of each time instance follows a temporal pattern, which indicates significant autocorrelation. Eq. (4) shows the autocorrelation of error $e_{t_{p}}$ with the errors $e_{t_{p}-1},\ldots,e_{t_{p}-d}$ of the previous *d*=12 time steps, since here one time step corresponds to 1 month.

Without autocorrelation, using Eq. (2), the error is minimized by achieving the optimal coefficient matrix *w* in linear regression model. With autocorrelation, as seen in Eq. (4), the new prediction error is reduced by using the vector of errors without autocorrelation from the past 12 months and multiplying it by the corresponding vector of coefficients (*ρ*_0_,…,*ρ*_*d*_) learned from the historical errors without autocorrelation.

**Fig. 4:**
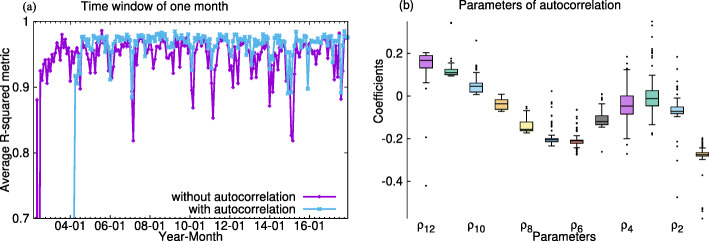
Autocorrelation correction for one-month predictions. **a** The fluctuations of performance are smaller for the predictions with autocorrelation for all lengths of historical data. **b** The coefficients of parameters of previous 12 months errors in the autocorrelation

In Fig. 4a, after applying autocorrelation, the prediction accuracy is increased by the decrease of correlated errors. Figure 4b shows that the error of the current month is correlated differently with the errors in the previous 12 months. The error is most positively correlated with the same month of the last year data, *ρ*_12_, while most negatively correlated with the last month, *ρ*_1_ and then the 6 months ago, *ρ*_6_. Figure 4b clearly shows seasonal change of the correlation.

### Predicting yearly per capita crime rate in community areas

For yearly predictions of per capita crime rate we use, as in the case of monthly predictions, the linear regression, selecting as features: *F*1, HomeOwnershipRate; *F*2, PovertyRate; *F*3, education level that combines HighschoolDegreeRate and BachelorDegreeRate; *F*4NeighborhoodCrimeRate, which is the average crime rate over all communities that are geographically adjacent to the given community; and, finally, *F*5CommunityCrimeRate.

We start by using all these features only for the last year before the prediction year. Then, we remove features that do not pass the null hypothesis test that requires *P*-value less than or equal to 0.05. As shown in Table 2, this yields features *F*5_1_ for immediate use and *F*4_1_ for later use because its p value is less than 0.1, where index of each feature indicates how many years before the forecast year are the data from which this feature value is computed. This set of features yields R-squared measure of 0.9338. Next, we add features for the second year before the forecast year and again preserve only those which survive the null hypothesis test. As shown in Table 2, the selected features are *F*4_1_,*F*5_1_,*F*4_2_, and they yield an excellent R-squared score of 0.9635. Adding the third year before the forecast features does not bring any improvement, so we keep the set of features *F*4_1_,*F*5_1_,*F*4_2_ as optimal.

**Table 2:** Performance of different set of features on predicting the crime per capita for 2017

Historical data depth	Coefficients	*P*-value	Coefficients	*P*-values	R-squared metric
1	*F*1_1_=−0.0017	*0.6358*	*F*5_1_=1.1786	<10^−55^	0.93
	*F*2_1_=−0.0016	*0.6087*			
	*F*3_1_=−0.0037	*0.5905*			
	*F*4_1_=−0.0888	0.0631			
	*F*5_1_=1.2198	<10^−47^			
2	*F*4_1_=0.7661	0.0002	*F*4_1_=0.8484	<10^−5^	0.96
	*F*4_2_=−0.8118	<10^−5^	*F*4_2_=−0.8955	<10^−6^	
	*F*5_1_=1.2432	<10^−20^	*F*5_1_=1.1181	<10^−89^	
	*F*5_2_=−0.1234	*0.2738*			
3	*F*4_1_=1.1177	<10^−9^			
	*F*4_2_=−1.0378	<10^−6^			
	*F*4_3_=−0.0702	*0.6889*			
	*F*5_1_=1.1967	<10^−28^			
	*F*5_2_=−0.2443	*0.0334*			
	*F*5_3_=0.0953	0.2526			

When deciding which feature to use for prediction with the current length of the historical data, we disregard features, printed in italics, that do not pass the test of null hypothesis that requires *P*-value less than or equal to 0.05 to avoid over-fitting. Therefore the feature sets containing such features are not assigned R-squared value. Before we increase the length of historical data, we retain features that pass less stringent test of *P*-value less than 0.1 to avoid losing the valid feature because of autocorrelation. Those features are printed in bold font. We stop when increasing the length of historical data does not improve R-squared metric for the model. To assess influence of each of the three features for the optimal historical data length of 2 years, we compute the normalized values of their coefficients that are as follows: *F*4_1_=0.0149,*F*4_2_=−0.0139 and *F*5_1_=0.0327, both F4 features have influence of about 40% of the F5 feature influence, but the former have opposite sign to each other which weakens their influence significantly

This result demonstrates that demographic characteristics of the community’s inhabitants are so-well encoded in last year crime rate of the given community area, *F*5_1_, and the average crime rate of its neighbors for the last year *F*4_1_, and the year before *F*4_2_, that direct use of the demographic data is not needed.

**Fig. 5:**
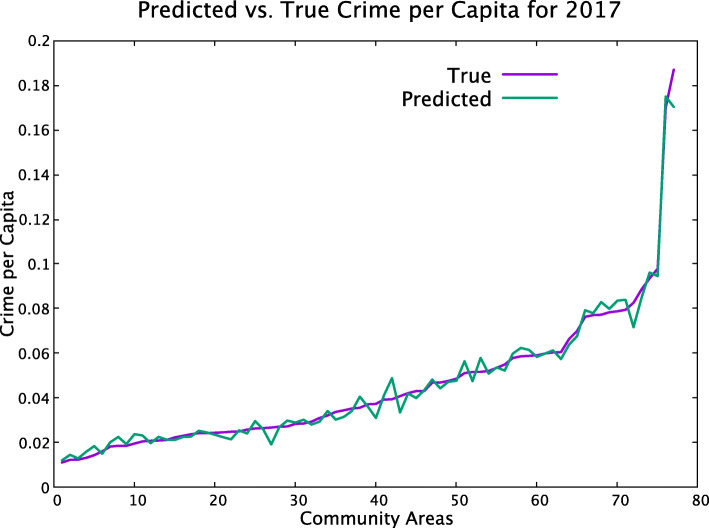
Forecasting the crime per capita rate for the upcoming year. The predictions are plotted versus true per capita crime rate for year 2017 using features *F*4_1_,*F*5_1_, and *F*4_2_, so using two years of data for feature *F*_4_. On the X-axis, community areas are listed in the increasing order of the crime rate per capita

Figure 5 shows comparison between the true per capita crime rate for the year 2017 using features *F*4_1_,*F*5_1_, and *F*4_2_. These results demonstrate that yearly predictions perform better than monthly ones. The reason is that the monthly data has twice as high standard deviation, when normalized by the average value, than yearly data does.

### Reducing city crime by additional crime reduction funding

#### Goals and approach

In this section we ask a simple but important question motivated by our results showing that crime rate in the given community is influenced by this rate in the communities that are geographical neighbors of this community. Deploying additional crime reduction funding, such as increasing home ownership, improving schools or, if inadequarte, increasing law enforcement personnel was shown to help. In (Weisburst 2018) the authors report that 10% increase in police employment rates reduces violent crime rates by 13%, and property crime rates by 7%. In (Mello 2019) the authors report that an additional police officer prevents 1.9 robberies and 5.1 auto thefts. Hence the question arises to which community or communities shall additional resources be deployed to obtain the largest reduction of crime in the entire city? We will refer to such deployment as an intervention. Since in reality we are not aware that such crime prevention funding had been deployed, we assume that no interventions were made in the period 2002–2017 which we research in this study. To be able to use our prediction methodology developed in the previous section, we make the following simplifying assumptions. First, we assume that interventions will be defined by their effects on crime in a community in which they are deployed. For the lack of the relevant data, we will not try to estimate what is the cost of such intervention or whether such cost is or is not dependent on the community of deployment. Moreover, we assume that size of crime reduction is small compared to the current crime level in the affected district.

In the linear regression the range of validity of its coefficients is limited. Yet, by their nature, these coefficients change a little if the operating point for simulation with intervention is close to the original operating point. Since we limit interventions to at most a few percent for only one of the 77 dimension at a time, the operating point with intervention is very close to the original operating point. Therefore quality of predictions in such a case deteriorates just a little. This approach enables us to claim that the models developed for each year of crime in the city are still valid when making predictions with crime reduced by an intervention.

We evaluate first theoretically and then by simulations the efficiency of a simple, one-year intervention. We experiment with the version in which all additional funding is deployed to one community. We do that by systematically choosing the community for deploying intervention and predicting city crime rate for the next year with crime reduced in this district by intervention. Our theoretical analysis show that more complex scenarios, like selecting several communities for one-year intervention, or doing interventions over a two year period cannot match the efficiency of the simple scenario.

For the one-year intervention, we use the most recent crime data for the year 2017 to compute effect of intervention. We use the most efficient model for prediction of crime in this year which uses crime crime rate as a feature F5 CommunityCrimeRate for the last year before the forecast and the spatial features F4 NeighborhoodCrimeRate accounting for crime in neighborhood communities for the last two years before the forecast. Then for each community chosen for intervention, we reduce its crime rate for year 2016 as indicated by the size of intervention.

#### Optimizing simple one-year one-community intervention

We use the following notation. Let *C*_*c*_(*t*) denote total number of crimes in the city in year *t*, *P*_*c*_(*t*) denote the population of the city in year *t*, and *d*_*n*_=77 stand for the number of community areas. Then, the average crime rate in the city is *C*_*c*_(*t*)/*P*_*c*_(*t*) and average population of the community is *P*_*c*_(*t*)/*d*_*n*_ so their product, *C*_*c*_(*t*)/*d*_*n*_, is the average number of crime for the average size community. We define the intervention size in terms of the number of crimes reduced by rate *r*=0.025 from the above value, so *V*_*r*_(*t*)=*rC*_*c*_(*t*)/*d*_*n*_. The initial number of crimes reduced in the community *d* is *I*_*d,r*_= min(*V*_*r*_()/*r*,1).

To compare the results, we compute the branching rate of crime change in community *d* as the crime reduction multiplier metric, denoted *M*_*d,r*_=*T*_*r*_/*V*_*r*_(*t*−1) for year *t*, where *T*_*r*_ denotes the total number of crimes in the city in year *t* under intervention with rate *r* in community *d* in year *t*−1.

Let’s consider an intervention in community *d* of size *I*, i.e., the number of crimes that this intervention should reduce in year *t*. There are three types of communities from the point of view the impact that this intervention makes.

1. Let’s denote by *ΔC*_*i,t*+1_ the number of crimes reduced by intervention in year *t*+1 community *i*. Because of presence of additional crime reduction funding in year *t*, the number of crimes in that year in community *i* decreases by *I*. Hence, the rate of crimes for this community changes from *C*_*i,t*_/*P*_*i,t*_, where *C*_*i,t*_ and *P*_*i,t*_ denote the number crimes and population community *i* in year *t*, to (*C*_*i,t*_−*I*)/*P*_*i,t*_. In year *t*+1,values for features *F*4_1_ and *F*4_2_ are the same as without intervention, since there is no change in the neighborhood of community *i* in years *t*−1,*t*, but the value for feature *F*5_1_ changes and therefore crime rate changes as follows.
  $$F5_{1}\left(\frac{C_{i,t}}{P_{i,t}} - \frac{C_{i,t}-I}{P_{i,t}}\right) = \frac{F5_{1}I}{P_{i,t}}. $$
  Hence, the actual change in the number of crimes is
  5 $$ \Delta C_{i,t+1} = F5_{1}I\frac{P_{i,t+1}}{P_{i,t}}.  $$
2. For any community *n* which is a neighbor of community *i* we have no change for value for features *F*5_1_ and *F*4_2_. The only change happens for the value of feature *F*4_1_ in year *t*.
  $$\Delta C_{n,t+1} = F4_{1}P_{n,t+1} \left(\frac{\sum_{k\in N_{n}}C(k,t)}{\sum_{k\in N_{n}}P(k,t)} - \frac{\sum_{k\in N_{n}}C(k,t)-I}{\sum_{k\in N_{n}}P(k,t)}\right) = F4_{1}I\frac{P_{n,t+1}}{\sum_{k\in N_{n}}P(k,t)}, $$
  where *N*_*n*_ denotes neighbors of community *n*.
3. Finally, for any other community that is neither *d* nor neighbor of *i* nothing changes in values of features for years *t*−1,*t* since neither there is a change for such a community nor for its neighbors, so there is no change of prediction for such community for year *t*+1.

Summing up the changes from cases 1 and 2, we get formula for *M*_*i,t*+1_ as

6 $$ M_{i,t+1} = F5_{1}\frac{P_{i,t+1}}{P_{i,t}} + F4_{1}\sum_{n \in N_{i}} \frac{P_{n,t+1}}{\sum{k\in N_{n}} P_{k,t}}   $$

Analyzing Eq. (6), it is clear that the branching rate of crime change does not depend on size of intervention *I*. Moreover, the first term is larger if the population of community *i* grows over time than when it declines. The second term is larger when the neighbors of community *i* are large compared to the population of their neighborhoods. Finally, it is clear that when intervention is spread over the years, any reduction of number of crimes in year *t*−1 will have negative effect for reduction of crime in year *t*+1 because coefficients of features *F*4_1_ and *F*4_2_ have opposite signs.

We start generating results by measuring how reducing crime in a given community by *V*_*r*_(*t*) for just the year 2016 affects the overall crime observed in the whole city for the year 2017. One by one, we apply intervention to each community, and leave all the others unchanged to see which of the communities has the highest branching rate of crime reduction. The results shown in Table 3 list the three most influential community areas and their impact sorted in the decreasing order of their branching rate of crime reduction.

**Table 3:** The three most influential communities (in decreasing order) for the year 2016 are 62, 4, and 29. Also shown are the numbers of crimes reduced in the community with intervention, and in the entire city

Community ID	*V*_*r*_(*t*)	*M*_*d,r*_	Number of crimes reduced in community with intervention	Number of crimes reduced in the city
62	37.64	2.737	42.22	103.03
4	37.64	2.685	42.88	101.07
29	37.64	2.542	42.05	95.70

The complementary Table 4 shows the three least influential communities, that have the branching rate of crime change lower by 50% between the best and the worst community, but all communities increase the initial intervention reduction in the very next year.

**Table 4:** The three least influential communities (in the increasing order of their branching rate of crime reduction) for the year 2016 are 74, 56, and 30. We look at how deploying interventions to different communities impacts the 2017 reduction in the number of crimes in the entire city

Community ID	*V*_*r*_(*t*)	*M*_*d,r*_	Number of crimes reduced in community with intervention	City wide crime reduction in 2017
74	37.64	1.378	42.52	51.86
56	37.64	1.439	42.82	54.19
30	37.64	1.554	42.69	58.51

#### Conclusions, limitations and future work

In this section we established that some communities are much more influential reducing crime than others. The most influential community, 62, has the branching rate of crime reduction of about 2.74, while the least influential community, 29, has this value less than 1.38, about just 1/2 of the value for the community 62. This means that any intervention needs to be carefully planned.

One limitation of our work is that the city wide crime reductions are based on predictions, with no means in our disposal to test those predictions in reality. This means that interventions need to be small enough to preserve validity of the model. Fortunately, our goal is to identify the most influential community which requires just getting the order of communities in terms of their crime reduction ability right. Since order of the results is more resistant to their errors than absolute values are, the results presented here could be useful.

### Classifying crimes by category

Predicting accurately the crime rate in a city community helps law enforcement agencies to prepare and plan for reducing city crime as inform well as inhabitants and visitors planning their travels there about the types of crimes to expect there. Moreover, gaining insight not only about the crime rate forecast for a city community, but also on the crime category to which the reported incidents of crime belong, can further facilitate more sophisticated crime-specific prevention strategies (Perry et al. 2013). At the minimum, it can suggest what category of crime will be prevalent in the given time frame, season and neighborhood.

The Chicago Police Department’s Illinois Uniform Crime Reporting (IUCR) code (Chicago Police Department) identifies 11 violent and property crime categories for the City of Chicago. Two of these, Ritualism and Offense Involving Children, occur so rarely throughout the studied time period that we decided to focus our analysis on the remaining 9 crime categories. Facing this multinomial (multiclass) problem, we study how accurately we can classify reported incidents of crime into the correct crime category. We studied the following four supervised machine learning algorithms: k-nearest neighbors (kNN), decision tree (DT), Naive Bayes (NB) and Support Vector Machine (SVM) and analyzed their performance with respect to various feature selections and crime categories. For these algorithms, initially we used the same features (date, community area, number of incidents) as for the one month linear regression analysis, but also included a true value using the data PrimaryCategory to label each incident with a crime category.

**Fig. 6:**
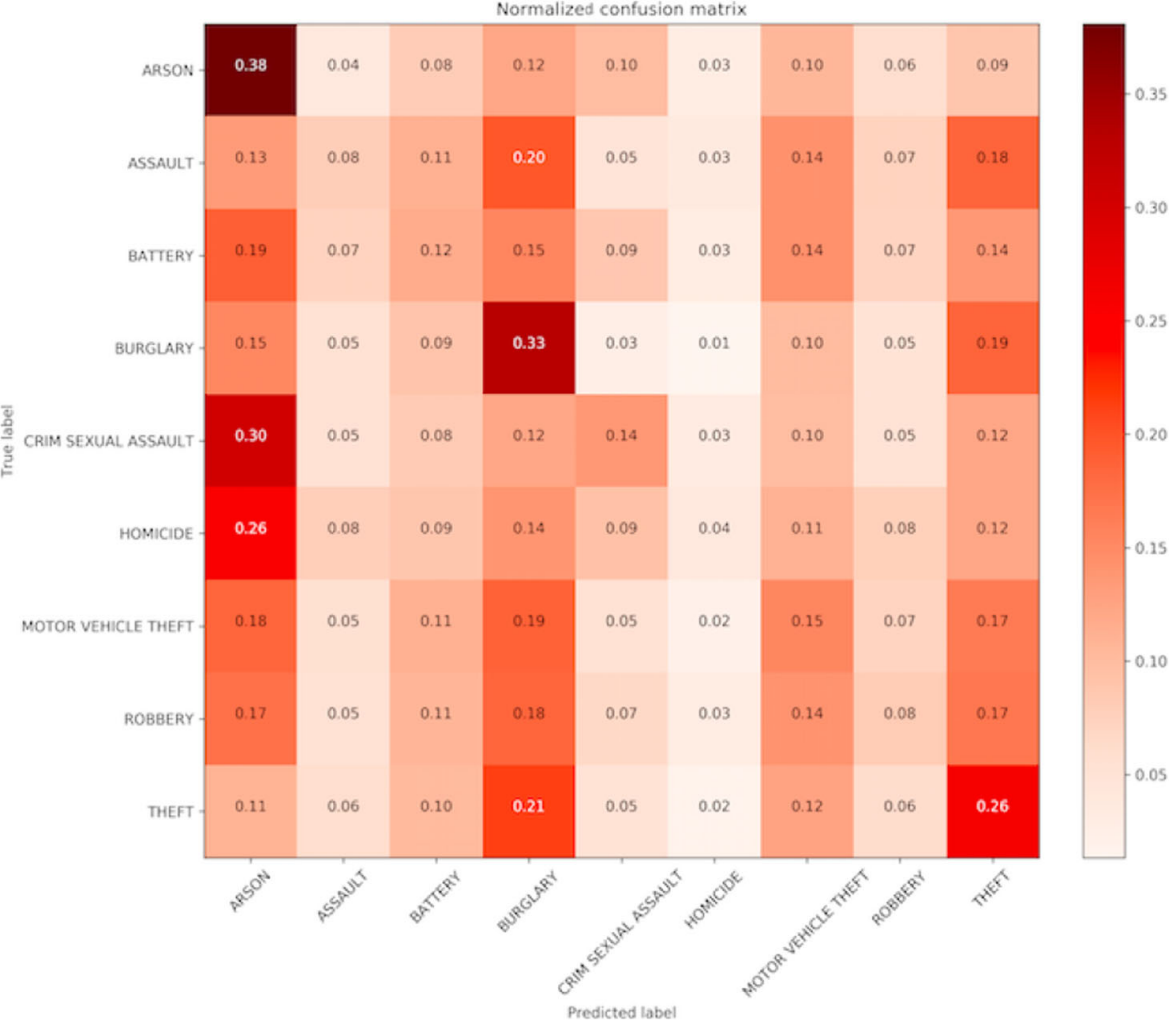
Confusion matrix of Naive Bayes classifier. The matrix plots the performance of the classifier in correctly labeling each crime category

**Fig. 7:**
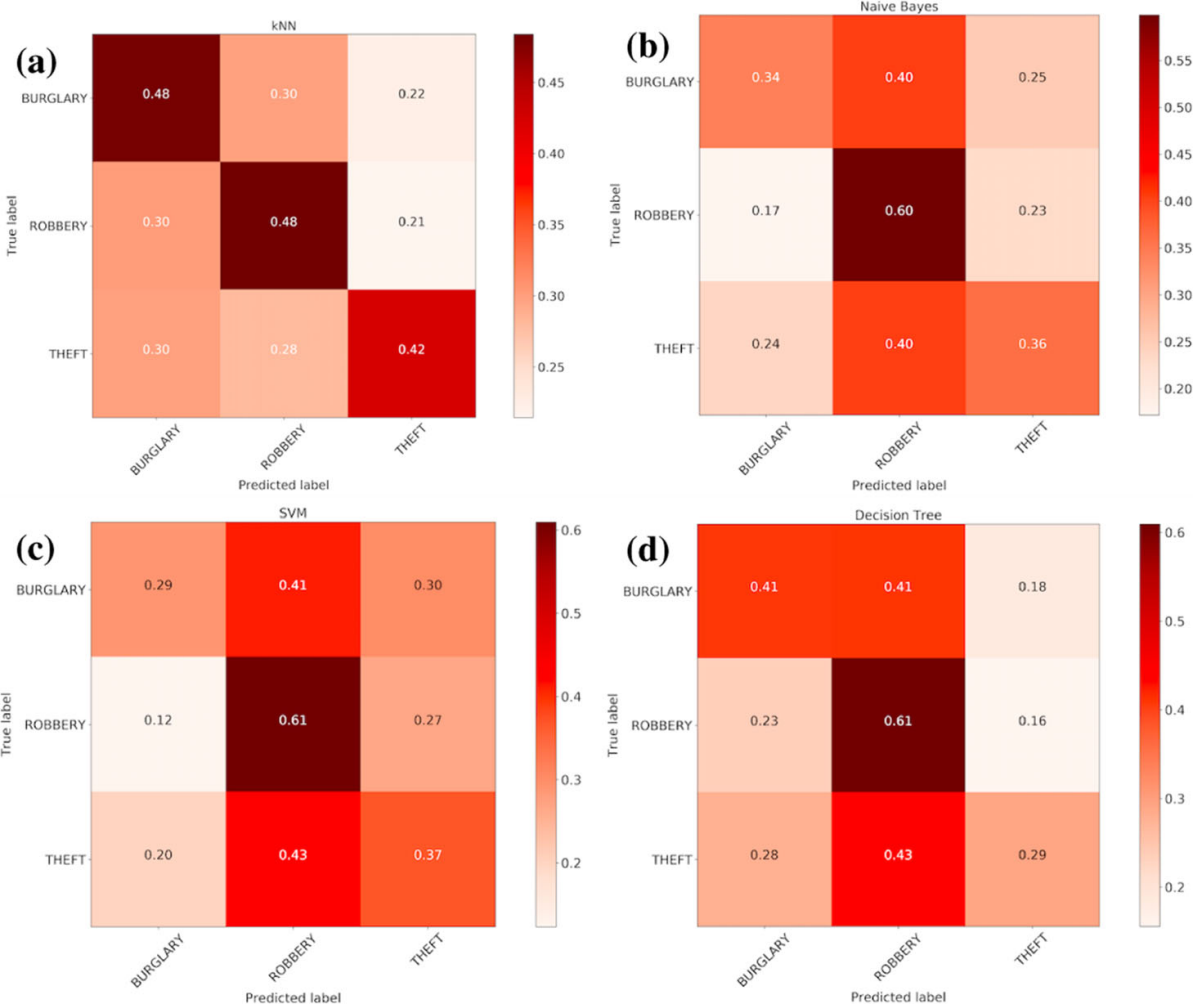
Confusion matrix for performance analysis of four different classification algorithms. **a** kNN (k-nearest neighbors), **b** Naive Bayes, **c** SVM (support vector machine) and **d** decision tree algorithms for classifying crime incidents into the top three most frequent crime categories occurring in the City of Chicago

We find that using only the minimal number of features, Date and Location from available data, we cannot label crime incidents with higher than 20% accuracy. Thus, we generated also the additional features: Year, Month, DayofWeek (range: Monday - Sunday), Weekend (range: yes no), TimeofDay (range: morning, afternoon, evening, night). By including information about the time of day and the day of week when the crime occurred, we increased the accuracy of correctly labeling crime incidents from 20% up to 50%. In Fig. 6 we plot the confusion matrix for the kNN algorithm, which demonstrates that by incorporating these temporal information in our classification algorithm, we can further increase the overall classification power, and we can correctly label arson, burglary and theft with the high accuracy compared to other crime categories. Lastly, in Fig. 7 we compare the performance of kNN (k-nearest neighbors), decision tree, Naive Bayes and SVM algorithms in labeling crime incidents into the correct class. For comparison purpose, we extract for this analysis the top three most frequently occurring crime categories in the City of Chicago, and plot the confusion matrices. We find that each method can classify with the highest and similar accuracy the Robbery crime category. The figures also reveal that the kNN classifier is the most robust among the four algorithms, with high accuracy in correctly labeling each crime category.

It is important to explain that our motivation for building the classifiers was not to develop a strong classification algorithm, but to demonstrate that by taking into account the temporal characteristics (time of a day, day of the week, etc.) of crime incidents, we can substantially improve the model predictive capabilities. This finding further proves that high versus low crime communities exhibit different temporal dynamics, and modeling should be sensitive to these different patterns. As pointed out in (Almanie et al. 2015), the results of these patterns could be used to raise people’s awareness regarding the dangerous locations and to help agencies to predict future crimes in a specific location within a particular time.

## Conclusions

Our first contribution here is uncovering correlations between criminal activities in the given community, and the demographic characterization of this community inhabitants. We reveal existence and dynamics of the criminal hot-spots in the City of Chicago in community areas monitored over 16 years of data collection. We show that communities characterized by high criminal activities exhibit different crime behavioral patterns than the ones experiencing low number of crime incidents. Additionally, we have revealed the demographic landscape of these areas and identified features such as high poverty rate, and lack of property ownership as strongly correlated with high crime community areas. Using linear regression to predict criminal activities, we establish the limits of predictive capabilities for forecast for that month number of crimes in the given community based on the number of crimes for one month from historical data. We find that it is possible to accurately forecast the number of crime incidents for the upcoming month from past crime data longer than 12 months. However, if the historical data is shorter, the prediction accuracy is low, unless features derived from the demographic data are added.

For accurate yearly predictions of crime rates per capita, we started with five features: *F*1HomeOwnershipRate, *F*2EducationLevelScore, *F*3PovertyRate, related to demographic data, and *F*4NeighborhoodCrimeRate, and *F*5CommunityCrimeRate for the given community area. Further, analysis reveals that the last two features are by far the most important. Moreover, to get the best results, two years of historical data are needed for feature *F*4 but only one year for feature *F*5.

We also study how reducing crime in a community by supporting additional crime reduction funding in this community impacts the crime in the entire city. We find that selection of the right community for deployment is important for achieving the highest branching rate of crime change.

Lastly, we analyze classification capabilities of correctly labeling criminal activities with the appropriate violent crime category. We find that by using exclusively historical information provided by the current data set (date and location), does not yield a strong classification algorithm. However, we showed that by generating additional features that capture a more detailed information about the time when the crime occurs (day of week and time of day), significantly improve our classification algorithm. Moreover, we find that using such information, certain crime categories (i.e. arson, burglary, theft) can be forecast with high accuracy. As pointed out in (Almanie et al. 2015), the discovered patterns could be used to raise people’s awareness regarding the level of crime and their types locations and to help agencies to predict future crimes in a specific location within a particular time.

## Data Availability

All data used in this article is publicly available at the websites cited in the references. Program source code described in the paper will be made available to any interested parties by contacting corresponding author.

## Abbreviations

CLEAR: Citizen law enforcement analysis and reporting system
IUCR: Illinois uniform crime reporting
CTA: Collaborative technology alliance **NS**: network science
kNN: K-nearest neighbors **NB**: naive bayes **DT**: decision tree
ONR: The office of naval research **SVM**: support vector machine
U.S.: The United States of America
